# Cerebral amyloid angiopathy in spontaneous intracerebral hemorrhage – radiology and immunohistochemistry in a consecutive surgical series

**DOI:** 10.1016/j.bas.2025.105884

**Published:** 2025-11-23

**Authors:** Ralf Watzlawick, Roland Roelz, Leon-Shanun Schroth, Ahmed Elbaz, Marc Hohenhaus, Mateo Tomas Fariña Nuñez, Jakob Straehle, Samer Elsheikh, Marco Prinz, David Seiffge, Daniel Erny, Mukesch Shah, Urs Fischer, Jürgen Beck

**Affiliations:** a Department of Neurosurgery, Medical Center - University of Freiburg, Faculty of Medicine, University of Freiburg, Freiburg, Germany; b Department of Neuroradiology, Medical Center - University of Freiburg, Faculty of Medicine, University of Freiburg, Freiburg, Germany; c Department of Spine Surgery, Schulthess Klinik, Zurich, Switzerland; d Center for Advanced Surgical Tissue Analysis (CAST), Faculty of Medicine, University of Freiburg, Freiburg, Germany; e Institute of Neuropathology, Medical Faculty, University of Freiburg, Freiburg, Germany; f CIBSS Centre for Integrative Biological Signalling Studies, University of Freiburg, Germany; g Department of Neurology, Inselspital Bern University Hospital and University of Bern, Bern, Switzerland

**Keywords:** Intracerebral hemorrhage, Stroke, Radiological score, Small vessel diseases, Cerebral amyloid angiopathy

## Abstract

**Introduction:**

Cerebral amyloid angiopathy (CAA) is a major cause of spontaneous intracerebral hemorrhage (ICH) in elderly patients and is associated with a high risk of recurrent hemorrhagic events.

**Research question:**

Whether the likelihood of CAA can be estimated using radiological criteria such as the Edinburgh criteria.

**Material and methods:**

We conducted a retrospective analysis of consecutive patients with spontaneous ICH who underwent neurosurgical hematoma evacuation between 01/2023 and 02/2025. Baseline clinical characteristics, radiological findings, and histopathological evaluations were assessed. Patients were classified as CAA-positive or CAA-negative based on immunohistochemical staining for amyloid-β deposition from intraoperative biopsies.

**Results:**

53 patients met the inclusion criteria and CAA-positive patients were significantly older (76.9 vs. 65.4 years, *p* < 0.001). Infratentorial hemorrhages were exclusively observed in the non-CAA group (*p* < 0.01). The presence of irregular ICH borders (*p* < 0.05) and concurrent subarachnoid hemorrhage (*p* < 0.001) were more common in CAA patients. Established radiological criteria for CAA could not predict the histopathological results for all patients. The diagnostic accuracy of the radiological simplified Edinburg criteria was acceptable (AUC: 0.76, 95 % CI: 0.61–0.92). However, according to these CT-based criteria, from all immunohistochemically CAA-negative patients (n = 13) 15.4 % (n = 2) were classified as having a high probability of CAA.

**Discussion and conclusions:**

Histopathologically confirmed CAA is associated with distinct radiological features, including ICH volume, irregular ICH borders and concurrent subarachnoid hemorrhage. Our findings highlight the importance of integrating histopathological analysis to improve diagnostic accuracy, as radiological criteria alone could not definitivey diagnose CAA in all patients.

## Highlights

• Cerebral amyloid angiopathy (CAA) is a major cause of spontaneous ICH.
• This retrospective study identified 53 ICH patients undergoing surgery.
• Intraoperative biopsies confirmed the histopathological diagnosis of CAA in 42 %.
• Established radiological criteria misclassified 15.4 % (n = 2) of all CAA-negative patients (n = 13).
• Accurate CAA-diagnosis is crucial and may alter further therapy after ICH.

## Abbreviations and acronyms

ICH: Intracerebral hemorrhage
SVD: Cerebral small vessel diseases
DPA: Deep perforator arteriolosclerosis
CAA: Cerebral amyloid angiopathy
CT: Computed tomography scan
MRI: Magnetic resonance imaging
mRS: Modified Rankin Scale
IQR: Interquartile range
CI: Confidence intervals
n: Number of patients
SE: Standard error
cc: Cubic centimeter [cm^3^]

## 1 Introduction

Intracerebral hemorrhage (ICH) constitutes up to 27 % of all global strokes and is associated with a high mortality rate and poor long-term functional outcomes (Collaborators, 2021; Qureshi et al., 2009; Seiffge et al., 2024). The majority (75 %–80 %) of non-traumatic ICH are attributable to cerebral small vessel diseases (SVD). Other potential causes of ICH include macrovascular lesions such as arteriovenous malformations and fistulas, aneurysms, cavernous malformations, and cerebral venous sinus thrombosis.

There are several forms of SVD, including rare monogenic SVDs such as cerebral autosomal dominant arteriopathy with subcortical infarcts and leukoencephalopathy (CADASIL). However, the clinically most pertinent major types of SVD are deep perforarrtor arteriolosclerosis (DPA) and cerebral amyloid angiopathy (CAA) (Seiffge et al., 2024; Goeldlin et al., 2022). DPA is the most common SVD form, claimed to account for over 90 % of non-lobar ICH. In lobar ICH, underlying SVD type is heterogenous with 40 % attributable to DPA alone, 20 % to CAA alone and 40 % to a mix of CAA and DPA (Rodrigues et al., 2018).

Identifying the underlying cause of ICH is important to determine prognosis and risk of ICH recurrence. CAA harbours a high risk of recurrence (up to 7.4 % in the first year) and was recently identified to be associated with early and locally adjacent recurrent ICH (Goeldlin et al., 2025).

The gold standard for diagnosis of CAA is postmortem full brain histopathological analysis (Goeldlin et al., 2022; Holling et al., 2012) using the Greenberg and Vonsattel grading system for CAA (Vonsattel et al., 1991), based on the extent of amyloid deposition in the vessel wall (Poyuran et al., 2019). Recent publications have required Vonsattel grade ≥1 (presence of amyloid in a vessel wall) to confirm the CAA diagnosis (Charidimou et al., 2022). Brain biopsies from patients may be acquired with the limitation of potential sampling error (Greenberg and Vonsattel, 1997) and only a small number of patients undergoing hematoma evacuation receive intraoperative biopsies. To overcome the limited availability of histopathological analysis, imaging-based classification for CAA have been developed: CAA can be diagnosed using acute computed tomography (CT) based on the Edinburgh criteria (Rodrigues et al., 2018) or magnetic resonance imaging (MRI) following the Boston 2.0 criteria (Charidimou et al., 2022). One limitation of the Edinburgh CAA criteria is that they were derived from post-mortem histopathological samples of patients deceased from ICH. It remains unclear how the criteria perform in patients who survive ICH. Additionally, several classification systems for ICH subtypes have been described, including CLAS-ICH (Raposo et al., 2023) and CADMUS (Goeldlin et al., 2024). However, a major limitation of all classification systems is the lack of histopathological validation (Seiffge et al., 2024).

The prevalence of CAA in ICH patients varies among different studies, primarily due to the varying diagnostic criteria employed. Only a few studies have examined histopathological tissue, predominantly in postmortem brain tissue, and reported a prevalence of CAA in ICH cases ranging from 9.1 % to 42 %. CAA diagnosis based solely on radiological assessment yielded a prevalence of 12 % in the National Taiwan University Hospital Stroke Registry, which included 4578 ICH patients (Poyuran et al., 2019; Yeh et al., 2014), and 20 % in a Helsinki study involving 1013 ICH patients (Poyuran et al., 2019; Meretoja et al., 2012). A meta-analysis conducted in 2022 (Jakel et al., 2022) revealed comparable proportions of probable CAA based on either histopathological or radiological diagnosis (according to the Boston criteria): 20–24 % of patients with ICH and 50–57 % of patients with lobar ICH. However, an unresolved challenge in small vessel disease (SVD) classification is distinguishing true mixed pathology of CAA and DPA from widespread DPA affecting both deep and lobar vessels (Seiffge et al., 2024). Evidence from a 2023 MRI-pathology study (Perosa et al., 2023) suggests that strictly lobar cerebral microbleeds may, in some cases, result from arteriolosclerosis. A recent study (Maury et al., 2025) evaluated the diagnostic performance of radiological criteria based on CT and MRI of 66 ICH patients within a 20 years inclusion period. This retrospective study of nonautopsied patients suggested that established radiological markers have lower diagnostic performance than previously reported. This underscores the imperative for histopathological confirmation of CAA in ICH patients.

In this study, we aimed to compare CT-based diagnosis of CAA using the Edinburgh CAA criteria with histopathological analysis in a cohort of patients with lobar ICH undergoing minimally invasive hematoma evacuation with biopsy.

## 2 Methods

This article conforms to the STARD reporting guideline.

### 2.1 Selection of patients

Patients with spontaneous ICH treated at the Department of Neurosurgery, Medical Center - University of Freiburg between 01/2023 and 02/2025 were identified. The study was approved by the local institutional ethics committee (University of Freiburg, No. 24-1157-S1). Patient consent was neither required nor sought due to the retrospective data collection in this consecutive series. We included patients with spontaneous, non-traumatic supratentorial ICH documented by CT scan, exclusion criteria were i) missing histopathological tissue, ii) macrovascular lesions and iii) missing immunohistochemical reaction. All patients were treated according to institutional protocols and ICH guidelines (Steiner et al., 2014) including immediate reversal of anticoagulant therapy and control of the systolic blood pressure. Standard procedures within the department did not include a prior documented diagnosis of CAA as a variable affecting the treatment decision.

### 2.2 Clinical data collection

Baseline clinical characteristics were extracted from available electronic patient records including preexisting medical conditions, drug intake and the first neurological assessment at admission (Table 1) by one reviewer (RW). Furthermore, the clinical course and treatment were retrospectively collected and managed using REDCap electronic data capture tools hosted at the local institution.

**Table 1:** Clinical baseline characteristics and comorbidities.

Variable	Overall (n = 53)	Patients without CAA (n = 31)	Patients with CAA (n = 22)	p-value
Age: years, median (IQR)	68.6 (61.4–78.2)	65.4 (58.1–72.2)	76.9 (68.9–83.0)	<0.001∗∗∗
Female sex, n/total	24/53 (45.3 %)	14/31 (45.2 %)	10/22 (45.5 %)	1.0
History of previous ICH, n/total (%)	3/53 (5.7 %)	0/31	3/22 (13.6 %)	0.13
History of previous stroke, n/total (%)	6/53 (11.3 %)	2/31 (6.5 %)	4/22 (18.2 %)	0.38
Arterial hypertension, n/total	33/53 (62.2 %)	20/31 (64.5 %)	13/22 (59.1 %)	0.48
Diabetes mellitus, n/total (%)	2/53 (3.8 %)	2/31 (6.5 %)	0/22	0.24
Renal insufficiency, n/total (%)	0/53	0/31	0/22	n.a.
Intake of hyperlipidemia medication, n/total (%)	14/53 (26.4 %)	5/31 (16.1 %)	9/22 (40.9 %)	0.18
Intake of antiplatelet therapy, n/total (%)	14/53 (26.4 %)	9/31 (29.0 %)	5/22 (22.7 %)	0.84
Intake of anticoagulants, n/total (%)	11/53 (20.8 %)	7/31 (22.6 %)	4/22 (18.2 %)	0.96
Initial systolic blood pressure, mean (SE)	162.7 mmHg (4.3)	165.9 mmHg (6.6)	160.4 mmHg (5.7)	0.39
Initial diastolic blood pressure, mean (SE)	80.4 mmHg (2.4)	81.8 mmHg (3.2)	78.4 mmHg (3.7)	0.46
Presence of bilateral pupillary light reflex at admission, n/total (%)	6/53 (11.3 %)	2/31 (6.5 %)	4/22 (18.2 %)	0.34
Intubated at admission, n/total (%)	17/53 (32.1 %)	10/31 (32.3 %)	7/22 (31.8 %)	1.0
GCS at admission, median (IQR)	11 (3–13)	11 (3–13)	11 (5.5–13)	0.93
Time between radiological assessment and surgical therapy: mean (SE)	15.2 h (7.3)	22.4 h (12.7)	6.3 h (1.6)	0.40
Treatment in intensive care unit, n/total	52/53 (98.1 %)	30/31 (96.8 %)	22/22 (100 %)	1.0
Treatment duration in intensive care unit: median (IQR)	10.5 days (7–18.3)	11 days (7–19.8)	9.5 days (8–15.3)	0.58
Timepoint of discharge after admission: median (IQR)	14.6 days (9.4–19.2)	14.0 days (9.2–23.4)	14.9 days (9.5–18.7)	0.96
Modified Rankin scale (mRS) at discharge: median (IQR)	5 (4–6)	5 (4–6)	5 (4–6)	0.93

Baseline characteristics and comorbidities at the initial clinical examination on admission are listed for all patients and patients with and without CAA diagnosed in brain biopsies. Statistical tests for group differences were performed, using Mann-Whitney-tests for non-parametric data and Pearson Chi^2^ for categorial variables. Absolute numbers, frequencies, Interquartile range (IQR), and p-values are shown. Asterisks indicate statistical significance: ∗p < 0.05, ∗∗p < 0.01, ∗∗∗p < 0.001.

### 2.3 Surgical procedure

Patients’ heads were fixed in a Mayfield skull clamp in the operating room. Following craniotomy and durotomy, CT navigation-guided or ultrasound-guided corticotomy was performed. Brain access tissue and hematoma tissue were subsequently obtained for histopathological analysis. Subsequently, patients were transferred to the intensive care unit, and a postoperative CT scan was conducted. All patients received the same medical treatment after the surgical procedure according to institutional procedures and ICH guidelines for the monitoring and treatment of ICH (Steiner et al., 2014). This included postoperative subcutaneous antithrombotic prophylaxis beginning 72 h after the ICH and the continuation of antiplatelet or anticoagulation therapy three weeks after the ICH.

### 2.4 Radiological assessment

Baseline CT scans obtained before surgery were assessed. Three independent reviewers blinded for histopathological results assessed the CT scans (RW, LS, AE). ICH volume was calculated using abc/2 as described previously (Kothari et al., 1996). Intraclass correlations were calculated for individual scores of the CT measurements and radiological parameters. Intraclass correlation coefficients (ICC) less than 0.5 indicate poor reliability, between 0.5 and 0.75 moderate reliability, between 0.75 and 0.9 good reliability, and greater than 0.90 excellent reliability (Koo and Li, 2016). We calculated the mean of the measurements from two reviewers with highest ICC. Discrepancies for the radiological items between the first and second reviewer were evaluated by the third reviewer. All CT scans were reviewd for subarachnoid hemorrhage and finger-like projections (Rodrigues et al., 2018). The diagnosis of CAA according to the simplified Edinburgh CAA criteria was made if both markers were present. The simplified Edinburgh score (Rodrigues et al., 2018) has been developed specifically for lobar ICHs only. Therefore, patients with non-lobar ICHs were excluded from this analysis.

### 2.5 Histopathological assessment

Trained neuropathologists assessed brain tissue biopsies using routine haematoxylin-eosin and elastica van Gieson stainings for vessel morphology and immunohistochemical reaction against amyloid beta (6F/3D, Dako). The brain tissue biopsies were labelled as CAA-positive according to the Vonsattel grading ≥1 (presence of amyloid in a vessel wall) (Vonsattel et al., 1991). Histopathological assessors were unaware of the radiological and clinical findings.

### 2.6 Statistical analysis

Baseline characteristics were assessed in the overall patient cohort. Data were subsequently tested for normal distribution using the Shapiro-Wilk normality test. Statistical comparisons were performed between patients without CAA and patients with CAA (immunohistochemical absence or presence of amyloid beta in a vessel wall). Continuous variables were described using median and quartiles. The Mann-Whitney test was employed to assess differences between the groups. Categorical variables were presented as frequencies and percentages, and comparisons were conducted using the chi-square or fisher exact test, as appropriate. Diagnostic accuracy (accuracy, sensitivity, specificity, positive and negative predictive values) were calculated using the *caret* package in R. Receiver operating characteristic (ROC) curve analysis was conducted using the *pROC* and *plotROC* package in R. All statistical analyses were performed using R (R version 4.3.1). All statistical tests were 2-sided and the level of significance was 0.05.

## 3 Results

**Fig. 1:**
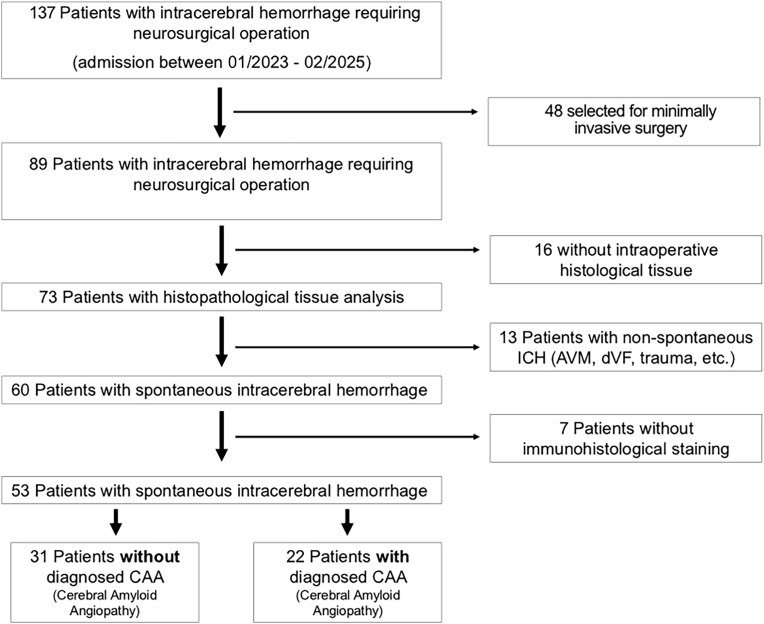
Patients selection flow-chart including eligibility criteria.

**Fig. 2:**
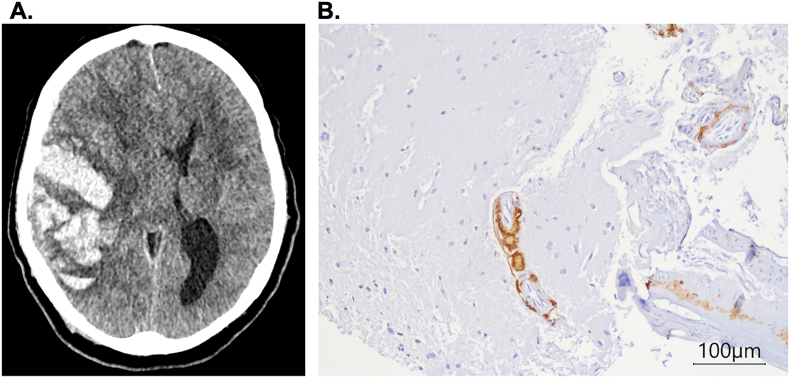
Immunohistological and radiological assessment Examples of CAA positive ICH in a 68 years old patient with **A.** the correlating CT scan showing ICH in atypical location and configuration and the **B.** deposits of Aβ partially/completely replacing the media.

Between 01/2023 and 02/2025 137 patients with spontaneous ICH were admitted to the Department of Neurosurgery, of whom 89 patients underwent operative hematoma removal via craniotomy (Fig. 1). Sixteen patients were excluded due to missing histopathological tissue from the surgical procedure. Additionally, 13 patients were excluded because of underlying macrovascular pathologies and 7 due to missing immunohistolochemistry. Consequently, 53 patients were included in the final analysis (Fig. 1). The median age on admission was 68.6 years (IQR: 61.4–78.2), and 24 patients (45.3 %) were female (Table 1). None of the patients had a previous documented CAA diagnosis before the ICH removal. The mean duration of stay for acute inpatient treatment was 17.9 days (SE: 1.93). Median modified Rankin scale score at discharge was 5 (IQR: 4–6). CT scans performed prior to ICH surgery were available for 84.9 % of all ICH patients (45/53 patients). Follow-up CT scans were available for all patients. Overall, 22 out of 53 patients (41.5 %) had a histopathologically confirmed diagnosis of CAA (Vonsattel grading ≥1) (see Fig. 2).

### 3.1 Clinical baseline characteristics and histological CAA confirmation

Patients with cerebral amyloid angiopathy (CAA) were significantly older compared to those without CAA (76.9 vs. 65.4 years, *p* < 0.001). There were no differences in preexisting medical conditions, medication intake or neurological examination between the two groups (Table 1). Histopathologically, additional amyloid plaques were exclusively found in 54.5 % of CAA-positive patients (12/22, p < 0.001).

### 3.2 Radiological parameters

Radiological rating of the initial CT imaging was performed by three raters and the intraclass correlation coefficient (ICC) was calculated. We observed a good reliability between the raters for ICH volume and midline shift, but only moderate and, in some instances, poor reliability for radiological parameters (Supplemental Table 1). Infratentorial ICH was exclusively observed in the non-CAA group (10/31 vs. 0/22, *p* < 0.01, Table 2). Lobar hemorrhages were more frequent in CAA patients (20/22 vs. 14/31), though this difference was not statistically significant. There was no significant difference in the affected hemisphere between the two groups (p = 0.19). CAA patients exhibited larger mean ICH volumes (82.5 ± 11.0 cc vs. 49.9 ± 6.0 cc, *p <* 0.05) and greater midline shift (7.4 ± 1.3 mm vs. 4.5 ± 1.0 mm, *p* = 0.08). The presence of irregular ICH borders (*p* < 0.05) and additional subarachnoid hemorrhage (*p* = 0.001) were significantly more common in the CAA group.

**Table 2:** Radiological assessment.

Variable	Overall (n = 53)	Patients without CAA (n = 31)	Patients with CAA (n = 22)	p-value
Supratentorial ICH	43 (81.1 %)	21/31 (67.7 %)	22/22 (100 %)	<0.01∗∗
Infratentorial ICH	10 (18.9 %)	10/31 (32.3 %)	0/22	
ICH Location –				0.19
right hemisphere	27/53 (50.9 %)	14/31 (45.2 %)	13/22 (59.1 %)	
left hemisphere	22/53 (41.5 %)	13/31 (41.9 %)	9/22 (40.9 %)	
both hemispheres	4/53 (7.6 %)	4/31 (12.9 %)	0/22	
Location of ICH – lobar	36/53 (67.9 %)	15/31 (48.4 %)	20/22 (90.9 %)	0.10
Location of ICH – deep	4/53 (7.5 %)	4/31 (12.9 %)	0	
Location of ICH – mixed	4/53 (7.5 %)	2/31 (6.5 %)	2/22 (9.1 %)	
ICH volume, mean (SE)	64.4 cc (6.4)	49.9 cc (6.0)	82.5 cc (11.0)	<0.05∗
Midline shift, mean (SE)	4.3 mm (0.8)	4.5 mm (1.0)	7.4 mm (1.3)	0.08
Presence of IVH	17/45 (37.8 %)	11/25 (%)	6/20 (%)	0.51
ICH irregular borders – yes	30/45 (66.7 %)	13/25 (%)	17/20 (%)	<0.05∗
ICH fingerlike projections – yes	23/45 (51.1 %)	10/25 (%)	13/20 (%)	0.17
Presence of additional subarachnoidal bleeding	28/45 (62.2 %)	9/25 (%)	19/20 (%)	<0.001∗∗∗
ICH variable density – yes	13/45 (28.9 %)	5/25 (%)	8/20 (%)	0.25

Initial native CT scans before surgery were assessed using the shown radiological criteria. Complete preoperative CT scans were available in 25/31 patients with CAA and 20/22 without CAA. Asterisks indicate statistical significance: ∗p < 0.05, ∗∗p < 0.01, ∗∗∗p < 0.001.

### 3.3 Simplified Edinburgh criteria for CAA detection based on CT imaging

Patients with lobar ICH only, available initial CT imaging and histopathological results were selected and the simplified Edinburgh score was calculated (31/36 all lobar ICH, Table 3). 18/31 (58.1 %) patients showed CAA-positive histology and the Edinburgh criteria indicated a low – medium – high probability of CAA for 1 (5.5 %) – 6 (33.3 %) – 11 (61.1 %) of these patients. Conversely, for 15.4 % of all CAA-negative patients (2/13 patients) the Edinburgh criteria indicated a high probability of CAA.

**Table 3:** Simplified Edinburgh score criteria for lobar ICH.

	Edinburgh criteria: Low probability of CAA	Edinburgh criteria: Medium probability of CAA	Edinburgh criteria: High probability of CAA
All patients (n = 31)	5 (16.1 %)	13 (41.9 %)	13 (41.9 %)
Histology: CAA negative (n = 13)	4 (30.8 %)	7 (53.9 %)	2 (15.4 %)
Histology: CAA positive (n = 18)	1 (5.5 %)	6 (33.3 %)	11 (61.1 %)

Based on the initial CT scan, the established simplified Edinburgh criteria were applied to assess the probability of CAA in all patients with supratentorial lobar ICH and stratified by histologically CAA-positive or CAA-negative findings.

We conducted a ROC analysis to evaluate the performance of the simplified Edinburgh criteria for the detection of CAA-positive histology. The area under the ROC curve indicated an acceptable discrimination for diagnosing CAA (AUC: 0.76, 95 % CI: 0.61–0.92, Supplemental Fig. 1). However, the presence of additional subarachnoidal bleeding (AUC: 0.74, 95 % CI: 0.59–0.89) and fingerlike projections (AUC: 0.61, 95 % CI: 0.43–0.79) indicated a poorer performance (Supplemental Table 2). The Edinburgh criteria indicating medium and high probability of CAA were associated with higher ICH volumes (Supplemental Table 3).

## 4 Discussion

Within our consecutive study of ICH patients undergoing surgery histopathological analysis revealed the etiology of ICH during neurosurgical treatment. 22/53 samples were tested CAA-positive (41.5 %), 31/53 were tested CAA-negative (58.5 %). Established radiological criteria alone could not definitively diagnose CAA in all ICH patients. This highlights the necessity of comparing and integrating radiological and histopathological findings to enhance the understanding of the etiology of ICH.

### 4.1 Clinical and radiological findings

Our findings demonstrate that patients with CAA were significantly older at presentation, consistent with previous studies highlighting age as a major risk factor for amyloid-related vasculopathy (Qureshi et al., 2009; Seiffge et al., 2024; Goeldlin et al., 2022; Raposo et al., 2023). The radiological assessment revealed a strong association between CAA and lobar hemorrhage, while non-CAA cases more frequently exhibited deep or infratentorial ICH. These findings align with the pertinent literature suggesting that CAA predominantly affects cortical and leptomeningeal vessels, leading to lobar hemorrhagic events (Seiffge et al., 2024; Rodrigues et al., 2018; Charidimou et al., 2022; Goeldlin et al., 2024). Additionally, the increased prevalence of irregular hemorrhage borders and concurrent subarachnoid hemorrhage in the CAA group suggests a distinct bleeding pattern, potentially reflecting the fragile nature of amyloid-laden vessels.

One major challenge in SVD classification is distinguishing ‘mixed’ neuroimaging patterns of CAA and DPA from widespread DPA affecting both deep and lobar vessels (Seiffge et al., 2024). Recent MRI-pathology correlation studies suggest that strictly lobar microbleeds, a hallmark of CAA, may in some cases be caused by arteriolosclerosis (Perosa et al., 2023). The simplified Edinburgh CT criteria demonstrated a rule-in specificity of 100 % for the detection of moderate or severe CAA assessing the combination of subarachnoid hemorrhage and finger-like projections (Rodrigues et al., 2018). Although we observed a correlation between the results of the Edinburgh CT scoring and the immunohistological CAA analysis, there were cases where the classification was incorrect within the studied group of patients.

The diagnostic accuracy of CT based diagnosis of CAA compared to other radiological biomarkers like MRI or amyloid PET is currently being discussed in the literature (Schwarz et al., 2023). The Boston criteria v 2.0 for MRI might improve the accuracy of a CAA diagnosis with a stated specificity and sensitivity of 84.6 % and 74.8 % in the original publication (Charidimou et al., 2022). However, individuals with mixed lobar and non-lobar haemorrhagic lesions remained an unresolved challenge (Charidimou et al., 2022). In a recent study (Schwarz et al., 2023) the diagnostic accuracy of CT based diagnosis of CAA compared to MRI biomarkers indicated a specificity and sensitity of 87.2 % and 29.6 %, showing that CT biomarkers might help to rule-in probable CAA. Another study compared the accuracy of MRI and CT based CAA diagnosis compared to amyloid PET and found no difference between MRI and CT accuracy (Michiels et al., 2022). Furthermore, a study on Dutch-type hereditary CAA revealed a decreased sensitivity for the CT based biomarkers depending on the ICH volumes which might lead to misclassification for smaller ICH volumes (van Etten et al., 2020).

This underscores the necessity for histopathological examination of brain biopsies as the gold standard to accurately classify ICH etiology. The use of intraoperative brain tissue sampling in this study allowed direct confirmation of vascular amyloid deposition, overcoming the limitations of radiological criteria alone. However, biopsy sampling error remains a potential limitation, as amyloid pathology may be focal and not uniformly distributed.

### 4.2 Recurrence of intracerebral hemorrhage

Of all lobar intracerebral hemorrhages (ICH) examined in this study, 42 % were associated with a CAA diagnosis. This finding aligns with the recent literature suggesting a prevalence of CAA in lobar ICH of approximately 50 % (Seiffge et al., 2024).

ICH due to CAA consistently associates with a significantly elevated risk of ICH recurrence compared to DPA (7.4–8.5 % versus 1.0–1.7 % per year) (Seiffge et al., 2024; Goeldlin et al., 2024; Charidimou et al., 2017; Fandler-Hofler et al., 2023; Greenberg and van Veluw, 2024). The underlying SVD may also influence the risk of ischemic vascular events, including ischemic stroke. At a 3-month follow-up, the risk of ischemic stroke for those with deep ICH mostly due to DPA was 4.3 % compared to lacunar strokes, although this result did not reach statistical significance in a study published in 2023 (Goeldlin et al., 2023).

Histopathological confirmation of intraoperatively obtained tissue may reveal CAA, potentially influencing subsequent medical treatment decisions. Patients without CAA and deep ICH locations may benefit from early continuation of antiplatelet and anticoagulation therapy following intracerebral hemorrhage (Seiffge et al., 2024). Patients with histopathologically diagnosed CAA might be harmed if oral anticoagulation is resumed, particularly for vitamin K antagonists and direct oral anticoagulants (Connolly et al., 2009; Granger et al., 2011; Patel et al., 2011; Ruff et al., 2014).

## 5 Limitations and future directions

This study has several limitations. First, the retrospective design encounters weaknesses such as missing data and loss to follow-up, which could potentially introduce bias. The ICH cohort exclusively includes patients requiring surgical intervention, which mainly includes clinically severe ICH cases with significant ICH volumes, also introducing selection bias. Second, while histopathological confirmation enhances diagnostic accuracy, the sample size remains relatively small, limiting the generalizability of our findings. Third, biopsy sampling error remains a challenge, as focal amyloid deposition may be missed in tissue sections. Future studies should aim to integrate advanced imaging techniques, such as amyloid PET, to enhance CAA detection. Fourth, the interrater reliability between three rater was only moderate and partly poor for radiological parameters. However, this pinpoints the subjectivity of items like fingerlike-projections in CT imaging which might also affect the accuracy of established radiological scores. Additionally, larger, prospective studies incorporating long-term follow-up data could further clarify the clinical impact of CAA-related hemorrhages.

## 6 Conclusion

Histopathologically confirmed CAA is associated with distinct radiological features and ICH volume. While radiological criteria such as the simplified Edinburgh classifications remain valuable diagnostic tools, classification by imaging alone was not correct for all patients in this study and may not be correct for many patients. Histopathological validation seem to be essential for accurate classification.

Brain biopsy based histopathological diagnosis can improve correct etiological classification, the pathophysiological understanding and management of patients with ICH. We conclude to consider brain (access tissue) biopsy in all surgical cases.

## Ethics approval

The study was conducted according to the guidelines of the Declaration of Helsinki, and approved by the Institutional Ethics Committee of the Medical Center University of Freiburg (No. 24-1157-S1).

## Availability of data and material

The used dataset, details of the statistical analysis and study protocol will be made available from the corresponding author on reasonable request.

## Authors’ contributions

Conceptualization: R.W., U.F., J.B.; methodology: R.W.; data acquisition: R.W., R.R., A.E., M.T.F.N., M.S., L.S.; data validation: R.W., M.S., M.H.; manuscript draft and graphical visualization: R.W., D.E., manuscript review and editing: R.W., J.B., D.E., R.R., M.H., J.S., U.F., M.S., M.P., S.E., D.S.; continuous supervision: R.R., M.S., J.B. All authors have read and agreed to the published version of the manuscript.

## Funding

This study received no funding.

## Conflicts of interest

The authors declare no conflict of interest.

## References

[bib1] Charidimou A., Imaizumi T., Moulin S., Biffi A., Samarasekera N., Yakushiji Y., Peeters A., Vandermeeren Y., Laloux P., Baron J.C., Hernandez-Guillamon M., Montaner J., Casolla B., Gregoire S.M., Kang D.W., Kim J.S., Naka H., Smith E.E., Viswanathan A., Jager H.R., Al-Shahi Salman R., Greenberg S.M., Cordonnier C., Werring D.J. (2017). Brain hemorrhage recurrence, small vessel disease type, and cerebral microbleeds: a meta-analysis. Neurology.

[bib2] Charidimou A., Boulouis G., Frosch M.P., Baron J.C., Pasi M., Albucher J.F., Banerjee G., Barbato C., Bonneville F., Brandner S., Calviere L., Caparros F., Casolla B., Cordonnier C., Delisle M.B., Deramecourt V., Dichgans M., Gokcal E., Herms J., Hernandez-Guillamon M., Jager H.R., Jaunmuktane Z., Linn J., Martinez-Ramirez S., Martinez-Saez E., Mawrin C., Montaner J., Moulin S., Olivot J.M., Piazza F., Puy L., Raposo N., Rodrigues M.A., Roeber S., Romero J.R., Samarasekera N., Schneider J.A., Schreiber S., Schreiber F., Schwall C., Smith C., Szalardy L., Varlet P., Viguier A., Wardlaw J.M., Warren A., Wollenweber F.A., Zedde M., van Buchem M.A., Gurol M.E., Viswanathan A., Al-Shahi Salman R., Smith E.E., Werring D.J., Greenberg S.M. (2022). The Boston criteria version 2.0 for cerebral amyloid angiopathy: a multicentre, retrospective, MRI-neuropathology diagnostic accuracy study. Lancet Neurol..

[bib3] Collaborators G.B.D.S. (2021). Global, regional, and national burden of stroke and its risk factors, 1990-2019: a systematic analysis for the global burden of disease study 2019. Lancet Neurol..

[bib4] Connolly S.J., Ezekowitz M.D., Yusuf S., Eikelboom J., Oldgren J., Parekh A., Pogue J., Reilly P.A., Themeles E., Varrone J., Wang S., Alings M., Xavier D., Zhu J., Diaz R., Lewis B.S., Darius H., Diener H.C., Joyner C.D., Wallentin L., Committee R.-L.S. (2009). Investigators, Dabigatran versus warfarin in patients with atrial fibrillation. N. Engl. J. Med..

[bib5] Fandler-Hofler S., Obergottsberger L., Ambler G., Eppinger S., Wunsch G., Kneihsl M., Seiffge D., Banerjee G., Wilson D., Nash P., Jager H.R., Enzinger C., Werring D.J., Gattringer T. (2023). Association of the presence and pattern of MRI markers of cerebral small vessel disease with recurrent intracerebral hemorrhage. Neurology.

[bib6] Goeldlin M., Stewart C., Radojewski P., Wiest R., Seiffge D., Werring D.J. (2022). Clinical neuroimaging in intracerebral haemorrhage related to cerebral small vessel disease: contemporary practice and emerging concepts. Expert Rev. Neurother..

[bib7] Goeldlin M.B., Vynckier J., Mueller M., Drop B., Maamari B., Vonlanthen N., Siepen B.M., Hakim A., Kaesmacher J., Jesse C.M., Mueller M.D., Meinel T.R., Beyeler M., Clenin L., Gralla J., Z'Graggen W., Bervini D., Arnold M., Fischer U., Seiffge D.J. (2023). Small vessel disease burden and risk of recurrent cerebrovascular events in patients with lacunar stroke and intracerebral haemorrhage attributable to deep perforator arteriolopathy. Eur Stroke J.

[bib8] Goeldlin M.B., Mueller M., Siepen B.M., Zhang W., Ozkan H., Locatelli M., Du Y., Valenzuela W., Radojewski P., Hakim A., Kaesmacher J., Meinel T.R., Clenin L., Branca M., Strambo D., Fischer T., Medlin F., Peters N., Carrera E., Lovblad K.O., Karwacki G.M., Cereda C.W., Niederhauser J., Mono M.L., Mueller A., Wegener S., Sartoretti S., Polymeris A.A., Altersberger V., Katan M., Psychogios M., Sturzenegger R., Nauer C., Schaerer M., Buitrago Tellez C., Renaud S., Minkner Klahre K., Z'Graggen W.J., Bervini D., Bonati L.H., Wiest R., Arnold M., Simister R.J., Wilson D., Jager H.R., Fischer U., Werring D.J., Seiffge D.J., for Swiss Stroke Registry I., Investigators S. (2024). CADMUS: a novel MRI-Based classification of spontaneous intracerebral hemorrhage associated with cerebral small vessel disease. Neurology.

[bib9] Goeldlin M.B., Fandler-Hofler S., Pezzini A., Manikantan A., Rauch J., Hald S.M., Kristensen M.L., Obergottsberger L., Sembill J.A., Haupenthal D., Larsen K.T., Avramiotis N.S., Polymeris A.A., Periole C., Thiankhaw K., Rangus I., Puy L., Pasi M., Morotti A., Silvestrelli G., Giacalone G., Paciaroni M., Zedde M., Giorli E., Tassi R., Delgado-Romeu M., Fischer U., Volbers B., Hakim A., Z'Graggen W.J., Nolte C.H., Werring D.J., Raposo N., Engelter S.T., Kristoffersen E.S., Kuramatsu J., Gattringer T., Gaist D., Seiffge D.J., collaborators E. (2025). Location and timing of recurrent, nontraumatic intracerebral hemorrhage. JAMA Neurol..

[bib10] Granger C.B., Alexander J.H., McMurray J.J., Lopes R.D., Hylek E.M., Hanna M., Al-Khalidi H.R., Ansell J., Atar D., Avezum A., Bahit M.C., Diaz R., Easton J.D., Ezekowitz J.A., Flaker G., Garcia D., Geraldes M., Gersh B.J., Golitsyn S., Goto S., Hermosillo A.G., Hohnloser S.H., Horowitz J., Mohan P., Jansky P., Lewis B.S., Lopez-Sendon J.L., Pais P., Parkhomenko A., Verheugt F.W., Zhu J., Wallentin L., Committees A. (2011). Investigators, Apixaban versus warfarin in patients with atrial fibrillation. N. Engl. J. Med..

[bib11] Greenberg S.M., van Veluw S.J. (2024). Cerebral amyloid angiopathy. Stroke.

[bib12] Greenberg S.M., Vonsattel J.P. (1997). Diagnosis of cerebral amyloid angiopathy. Sensitivity and specificity of cortical biopsy. Stroke.

[bib13] Holling M., Jeibmann A., Fischer B.R., Albert F.K., Ebel H., Paulus W., Stummer W. (2012). Histopathological analysis of intracerebral hemorrhage: implications for clinical management. Acta Neurochir..

[bib14] Jakel L., De Kort A.M., Klijn C.J.M., Schreuder F., Verbeek M.M. (2022). Prevalence of cerebral amyloid angiopathy: a systematic review and meta-analysis. Alzheimer's Dement..

[bib15] Koo T.K., Li M.Y. (2016). A guideline of selecting and reporting intraclass correlation coefficients for reliability research. J. Chiropr. Med..

[bib16] Kothari R.U., Brott T., Broderick J.P., Barsan W.G., Sauerbeck L.R., Zuccarello M., Khoury J. (1996). The ABCs of measuring intracerebral hemorrhage volumes. Stroke.

[bib17] Maury A., Benzakoun J., Bani-Sadr A., Ter Schiphorst A., Reiner P., Hosseini H., Seners P., Pallud J., Oppenheim C., Calvet D., Charidimou A., Varlet P., Turc G., Baron J.C., Group H.S. (2025). Diagnostic accuracy of finger-like projections and subarachnoid hemorrhage for cerebral amyloid angiopathy: pathological validation from Lobar Hematoma evacuation or brain biopsy. Stroke.

[bib18] Meretoja A., Strbian D., Putaala J., Curtze S., Haapaniemi E., Mustanoja S., Sairanen T., Satopaa J., Silvennoinen H., Niemela M., Kaste M., Tatlisumak T. (2012). SMASH-U: a proposal for etiologic classification of intracerebral hemorrhage. Stroke.

[bib19] Michiels L., Dobbels L., Demeestere J., Demaerel P., Van Laere K., Lemmens R. (2022). Simplified Edinburgh and modified Boston criteria in relation to amyloid PET for lobar intracerebral hemorrhage. Neuroimage Clin.

[bib20] Patel M.R., Mahaffey K.W., Garg J., Pan G., Singer D.E., Hacke W., Breithardt G., Halperin J.L., Hankey G.J., Piccini J.P., Becker R.C., Nessel C.C., Paolini J.F., Berkowitz S.D., Fox K.A., Califf R.M., Investigators R.A. (2011). Rivaroxaban versus warfarin in nonvalvular atrial fibrillation. N. Engl. J. Med..

[bib21] Perosa V., Auger C.A., Zanon Zotin M.C., Oltmer J., Frosch M.P., Viswanathan A., Greenberg S.M., van Veluw S.J. (2023). Histopathological correlates of lobar microbleeds in false-positive cerebral amyloid angiopathy cases. Ann. Neurol..

[bib22] Poyuran R., Mahadevan A., Arimappamagan A., Nandeesh B.N., Nagappa M., Saini J., Narasinga Rao K.V.L., Chickabasaviah Y.T. (2019). Cerebrovascular pathology in cerebral amyloid angiopathy presenting as intracerebral haemorrhage. Virchows Arch..

[bib23] Qureshi A.I., Mendelow A.D., Hanley D.F. (2009). Intracerebral haemorrhage. Lancet.

[bib24] Raposo N., Zanon Zotin M.C., Seiffge D.J., Li Q., Goeldlin M.B., Charidimou A., Shoamanesh A., Jager H.R., Cordonnier C., Klijn C.J., Smith E.E., Greenberg S.M., Werring D.J., Viswanathan A. (2023). A causal classification system for intracerebral hemorrhage subtypes. Ann. Neurol..

[bib25] Rodrigues M.A., Samarasekera N., Lerpiniere C., Humphreys C., McCarron M.O., White P.M., Nicoll J.A.R., Sudlow C.L.M., Cordonnier C., Wardlaw J.M., Smith C., Al-Shahi Salman R. (2018). The Edinburgh CT and genetic diagnostic criteria for lobar intracerebral haemorrhage associated with cerebral amyloid angiopathy: model development and diagnostic test accuracy study. Lancet Neurol..

[bib26] Ruff C.T., Giugliano R.P., Braunwald E., Hoffman E.B., Deenadayalu N., Ezekowitz M.D., Camm A.J., Weitz J.I., Lewis B.S., Parkhomenko A., Yamashita T., Antman E.M. (2014). Comparison of the efficacy and safety of new oral anticoagulants with warfarin in patients with atrial fibrillation: a meta-analysis of randomised trials. Lancet.

[bib27] Schwarz G., Banerjee G., Hostettler I.C., Ambler G., Seiffge D.J., Ozkan H., Browning S., Simister R., Wilson D., Cohen H., Yousry T., Al-Shahi Salman R., Lip G.Y.H., Brown M.M., Muir K.W., Houlden H., Jager R., Werring D.J. (2023). MRI and CT imaging biomarkers of cerebral amyloid angiopathy in lobar intracerebral hemorrhage. Int. J. Stroke.

[bib28] Seiffge D.J., Fandler-Hofler S., Du Y., Goeldlin M.B., Jolink W.M.T., Klijn C.J.M., Werring D.J. (2024). Intracerebral haemorrhage - mechanisms, diagnosis and prospects for treatment and prevention. Nat. Rev. Neurol..

[bib29] Steiner T., Al-Shahi Salman R., Ntaios G. (2014). The European Stroke Organisation (ESO) guidelines. Int. J. Stroke.

[bib30] van Etten E.S., Kaushik K., van Zwet E.W., Voigt S., van Walderveen M.A.A., van Buchem M.A., Terwindt G.M., Wermer M.J.H. (2020). Sensitivity of the Edinburgh criteria for lobar intracerebral hemorrhage in hereditary cerebral Amyloid angiopathy. Stroke.

[bib31] Vonsattel J.P., Myers R.H., Hedley-Whyte E.T., Ropper A.H., Bird E.D., Richardson E.P. (1991). Cerebral amyloid angiopathy without and with cerebral hemorrhages: a comparative histological study. Ann. Neurol..

[bib32] Yeh S.J., Tang S.C., Tsai L.K., Jeng J.S. (2014). Pathogenetical subtypes of recurrent intracerebral hemorrhage: designations by SMASH-U classification system. Stroke.

