# Supplementary material for: Cerebral amyloid angiopathy in spontaneous intracerebral hemorrhage – radiology and immunohistochemistry in a consecutive surgical series
Source: Brain Spine. 2025 Nov 23;5:105884. doi: 10.1016/j.bas.2025.105884 (PMC12702237; doi:10.1016/j.bas.2025.105884)
Supplement: Multimedia component 1 [file mmc1.docx]

**Supplement**

**Supplemental Table 1: Interrater reliability**

| **Variable** | **Rater 1 / Rater 2** | **Rater 1 / Rater 3** | **Rater 2 / Rater 3** |
| --- | --- | --- | --- |
| ICH volume | 0.73  (0.56 – 0.84) | 0.59  (0.32 – 0.76) | 0.81  (0.64 – 0.9) |
| Midline shift | 0.83  (0.7 – 0.9) | 0.896  (0.82-0.94) | 0.84  (0.73 – 0.91) |
| Presence of IVH | 0.43  (0.1 – 0.67) | 0.69  (0.51 – 0.82) | 0.51  (0.19 – 0.71) |
| ICH irregular borders – yes | -1.9^-16^  (-0.15 – 0.19) | 0.47  (0.21 – 0.67) | 6.3^-16^  (-0.13 – 0.18) |
| ICH fingerlike projections – yes | 0.28  (0.003 – 0.51) | 0.47  (0.20 – 0.67) | 0.31  (0.01 – 0.55) |
| Presence of additional subarachnoidal bleeding | 0.22  (-0.05 – 0.47) | 0.34  (0.06 – 0.58) | 0.32  (0.03 – 0.56) |
| ICH variable density – yes | 0.34  (0.06 – 0.58) | 0.42  (0.15 – 0.64) | 0.49  (0.23 – 0.68) |

Three independent reviewers blinded for histopathological results assessed the CT scans on admission. Intraclass correlation coefficients (ICC) less than 0.5 indicate poor reliability, between 0.5 and 0.75 moderate reliability, between 0.75 and 0.9 good reliability, and greater than 0.90 excellent reliability. 95% confidence intervals are shown in parentheses.

**Supplemental Table 2: Diagnostic accuracy of simplified Edinburgh criteria**

|  | **Presence of fingerlike projections** | **Presence of additional subarachnoidal bleeding** | **Presence of fingerlike projections AND subarachnoidal bleeding** |
| --- | --- | --- | --- |
| **Sensitivity (True positive rate)** | 0.62 | 0.54 | 0.85 |
| **Specificity (True negative rate)** | 0.61 | 0.94 | 0.61 |
| **Positive predictive value** | 0.53 | 0.88 | 0.85 |
| **Negative predictive value** | 0.69 | 0.74 | 0.42 |
| **Accuracy (95% CI)** | 0.61 (0.42 – 0.78) | 0.77 (0.59 – 0.90) | 0.71 (0.52 – 0.86) |

The simplified Edinburgh criteria include the presence of fingerlike projections on CT scans or additional subarachnoidal bleeding. The presence of both is characterized as a high probability of CAA.

**Supplemental Table 3: Simplified Edinburgh score criteria for lobar ICH including ICH volume**

|  | **Edinburgh criteria:**  **Low probability of CAA** | **Edinburgh criteria:**  **Medium probability of CAA** | **Edinburgh criteria:**  **High probability of CAA** |
| --- | --- | --- | --- |
| All patients (n=31) | 5 (16.1%)  Volume: 55.4 cc (12.4) | 13 (41.9%)  Volume: 54.0 cc (7.8) | 13 (41.9%)  Volume: 92.1 cc (12.5) |
| Histology: CAA negative (n=13) | 4 (30.8%)  Volume: 64.8 cc (10.5) | 7 (53.9%)  Volume: 57.6 cc (10.5) | 2 (15.4%)  Volume: 59.9 cc (10.4) |
| Histology: CAA positive (n=18) | 1 (5.5%)  Volume: 17.9 cc (-) | 6 (33.3%)  Volume: 49.8 cc (12.7) | 11 (61.1%)  Volume: 97.9 cc (14.1) |

Based on the initial CT scan, the established simplified Edinburgh criteria were applied to assess the probability of CAA in all patients with supratentorial lobar ICH and stratified by histologically CAA-positive or CAA-negative findings. The Edinburgh criteria indicating medium and high probability of CAA were associated with higher ICH volumes.

**Supplemental Figure 1: Receiver operating characteristic (ROC) curve analysis**


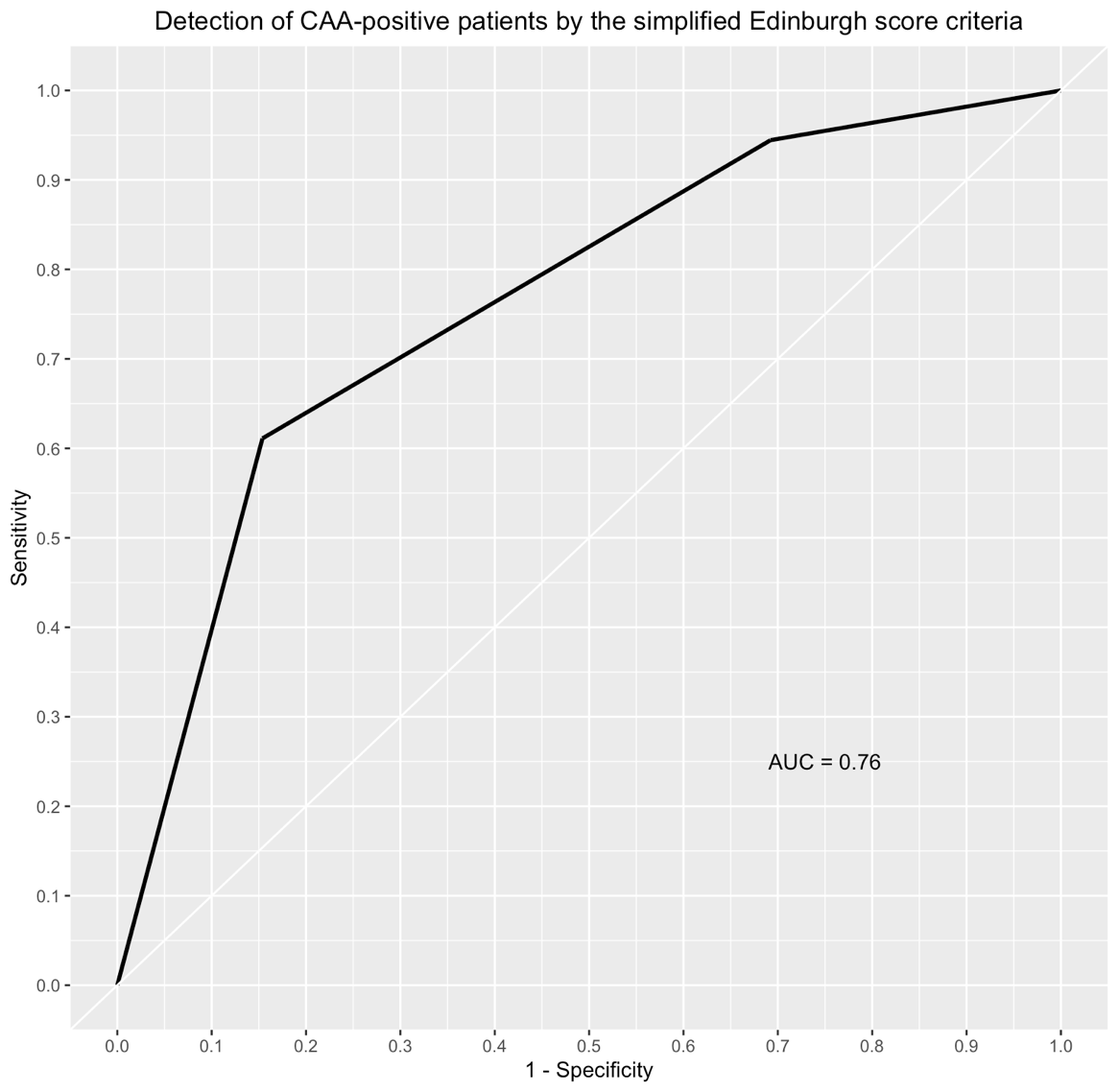


**Receiver operating characteristic curve analysis**. ROC analysis for the diagnosis of CAA by the simplified Edinburgh criteria, AUC 0.76 (95% CI: 0.61-0.92).
